# Long-Term Outcomes in Aortic Stenosis: Mortality Analysis in a Selected Patient Group

**DOI:** 10.3390/jpm15090410

**Published:** 2025-09-02

**Authors:** Olga Irtyuga, Mary Babakekhyan, Oleg Metsker, Anna Starshinova, Dmitry Kudlay, Georgy Kopanitsa

**Affiliations:** 1 Federal State Budgetary Institution “V.A. Almazov National Medical Research Centre” of the Ministry of Health of the Russian Federation, 197341 Saint-Petersburg, Russia; olgir@yandex.ru (O.I.); babakehyan97@mail.ru (M.B.); olegmetsker@gmail.com (O.M.); starshinova_aa@almazovcentre.ru (A.S.); 2 A.P. Nelyubin Institute of Pharmacy, M. Sechenov First Moscow State Medical University, 119571 Moscow, Russia; d624254@gmail.com; 3 Faculty of Fundamental Medicine, Lomonosov Moscow State University, 119234 Moscow, Russia; 4 Department of Pharmacology, Institute of Immunology FMBA of Russia, 115522 Moscow, Russia; 5 Laboratory of Digital Public Health Technologies, ITMO University, 197101 Saint-Petersburg, Russia

**Keywords:** aortic stenosis, gender differences, bicuspid aortic valve, survival, comorbidities

## Abstract

**Background:** Aortic stenosis (AS) is a prevalent acquired heart valve disease with increasing incidence, particularly among older adults. Gender-specific differences in AS presentation, comorbidities, and outcomes remain underexplored, necessitating further investigation to optimize personalized treatment strategies. **Objective:** To evaluate the clinical and demographic characteristics, comorbidities, and survival outcomes of patients with AS, stratified by gender and aortic valve morphology. **Methods:** A retrospective analysis of 145,454 echocardiographic examinations (2009–2018) at the Federal State Budgetary Institution “V.A. Almazov National Medical Research Centre” identified 84,851 patients meeting the inclusion criteria (Vmax ≥ 2.0 m/s, age ≥ 18 years). Patients were stratified by gender and valve morphology (bicuspid aortic valve [BAV] vs. tricuspid aortic valve [TAV]). Survival was assessed in 475 pts with AS over a 16-year period (2009–2025) using Kaplan–Meier analysis. Statistical comparisons utilized STATISTICA v. 10.0, with *p*-values derived from P-tests. **Results:** Of the cohort, 4998 men and 6322 women had AS. Men with AS were older (median 64 vs. 57 years, *p* < 0.0001) and had higher systolic blood pressure (140 vs. 130 mmHg, *p* < 0.0001) than men without AS. Women with AS were also older (median 70 vs. 58 years, *p* < 0.0001) with higher systolic (140 vs. 130 mmHg, *p* < 0.0001) and diastolic blood pressure (80 vs. 80 mmHg, *p* < 0.0001). Men with AS had higher rates of hyperlipidemia (HLP) (26.3% vs. 10.3%, *p* < 0.0001), while women with AS had increased coronary artery disease (CAD) (35.7% vs. 26.4%, *p* < 0.0001), diabetes mellitus (DM) (13.4% vs. 10.2%, *p* < 0.0001), and obesity (10.9% vs. 10.2%, *p* = 0.06). Chronic heart failure (CHF) was more frequently reported in patients with AS, regardless of gender, compared to patients without AS (in men 53.4% vs. 41.8%, *p* < 0.0001; in women 54.5% vs. 37.5%, *p* < 0.0001). BAV was associated with higher AS prevalence (54.5% in men, 66.4% in women). Survival analysis revealed higher mortality. Over the 16-year follow-up period, the mortality rate was 21.7%. **Conclusions:** Mortality in a representative AS cohort reached 21.7%, underscoring the progressive nature of the disease and its long-term impact. Survival was negatively affected by age over 68.5 years, as well as the presence of aortic regurgitation (AR), increased peak aortic jet velocity, and enlarged maximum aortic diameter. Aortic valve replacement demonstrates an insignificant effect on patient survival rates. Beta-blocker therapy in patients with varying degrees of aortic AS severity has not only demonstrated its safety but has also shown a positive effect on reducing mortality (improving survival). In contrast, the combination of angiotensin II receptor blockers (ARBs) with calcium channel blockers (CCBs) is quite dangerous for patients with AS and reduces their survival. Aortic valve replacement demonstrates an insignificant effect on patient survival rates. In contrast, the absence of fibrinolytic therapy and anticoagulant treatment is associated with an improved prognosis. Conversely, the administration of antiarrhythmic agents and statins is correlated with enhanced survival outcomes, potentially attributable to their influence on coexisting comorbidities. Further research is required to delineate their precise mechanisms and contributions. These results emphasize the importance of early identification, comprehensive risk assessment, and individualized management strategies in improving outcomes for patients with AS.

## 1. Introduction

AS represents one of the most prevalent acquired valvular heart diseases in adults, significantly contributing to cardiovascular morbidity and mortality worldwide [1,2]. Characterized by progressive narrowing of the aortic valve, AS imposes a substantial hemodynamic burden on the left ventricle, leading to heart failure, syncope, and sudden cardiac death if untreated [3,4]. Epidemiological data underscore a rising global burden of AS, with the Global Burden of Disease Study (GBD) reporting over 9 million cases of moderate to severe AS in 2019, and a prevalence exceeding 1000 cases per 100,000 among individuals aged over 75 years [5]. This increase is particularly pronounced in aging populations, driven by improved life expectancy and the growing incidence of degenerative calcific aortic valve disease, the predominant etiology of AS [6].

Calcific aortic valve disease, characterized by progressive leaflet calcification and fibrosis, accounts for most AS cases [7,8] in developed nations. The European Society of Cardiology reported a sevenfold rise in the detection of aortic valve calcification over the past three decades, with higher incidence rates in high-income countries, likely due to enhanced diagnostic capabilities and greater healthcare access [9]. Risk factors for calcific AS are well-established and include advanced age, male gender, genetic predisposition, arterial hypertension (HP), smoking, diabetes mellitus, obesity, elevated lipoprotein (a) levels, and BAV [8]. Notably, patients with BAV, a congenital anomaly present in 1–2% of the population, experience earlier onset and more rapid progression of AS compared to those with tricuspid aortic valves (TAV) [10].

Gender-specific differences in AS presentation, progression, and outcomes have garnered increasing attention in recent literature. Men are historically reported to have a higher prevalence of AS, potentially due to higher rates of risk factors such as hypertension and smoking [11,12,13]. However, women with AS exhibit distinct clinical profiles, including a lower likelihood of aortic valve calcification at the equivalent disease severity, smaller left ventricular dimensions, and higher ejection fraction (EF) values [14]. Women also tend to have fewer comorbidities compared to men with AS, yet paradoxically face lower five-year survival rates, possibly due to delayed diagnosis or differences in disease progression [11,12]. These disparities may be compounded by gender-specific responses to therapeutic interventions, such as transcatheter aortic valve replacement (TAVR), where women often experience better procedural outcomes but higher rates of complications like vascular injury [15].

The interplay of gender with AS pathophysiology underscores the need for a nuanced understanding of its clinical implications. Variations in hormonal profiles, genetic predispositions, and socioeconomic factors may further modulate disease expression and treatment responses between genders [11]. Consequently, elucidating gender-specific characteristics in AS is a critical research priority to inform personalized diagnostic and therapeutic strategies. This study aims to investigate the clinical and demographic profiles of patients with AS, stratified by gender, to enhance risk stratification and optimize management approaches for this increasingly prevalent condition.

## 2. Materials and Methods

The study was conducted in accordance with the principles of the Declaration of Helsinki. The protocol was approved by the local ethics committee of the Federal State Budgetary Institution “V.A. Almazov National Medical Research Centre”. №181 from 16 October 2023.

*Study Design*

**Figure 1:**
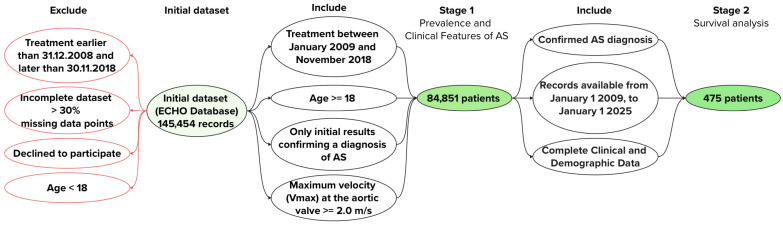
Study design.

The study consisted of two stages (Figure 1).

Stage 1

A retrospective analysis was conducted of 145,454 echocardiographic examinations performed from January 2009 to November 2018 at the Federal State Budgetary Institution “V.A. Almazov National Medical Research Centre”. Based on data completeness and inclusion criteria, 84,851 patients were selected for further analysis [16,17].

*Inclusion Criteria*

1. Patients treated between 1 January 2009, and 30 November 2018, who underwent transthoracic echocardiography (TTE) at the Federal State Budgetary Institution “V.A. Almazov National Medical Research Centre”;
2. Patients with a maximum velocity (Vmax) across the aortic valve ≥ 2.0 m/s;
3. Age ≥ 18 years at the time of inclusion;
4. Patient consent for participation in the study.

*Exclusion Criteria*

1. Patients under 18 years of age at the time of inclusion;
2. Patients with insufficient data for further analysis.

Extended clinical and demographic characteristics, including those stratified by aortic valve morphology, were previously reported in our earlier publications [16,17].

*Propensity Score Matching*

To adjust for baseline differences between AS and non-AS groups, we performed post-hoc 1:1 propensity score matching. Propensity scores were estimated via logistic regression with the following covariates: age, sex, hypertension, diabetes mellitus, coronary artery disease, atrial fibrillation, and heart failure. Nearest-neighbor matching without replacement was used with a caliper width of 0.2 standard deviations of the logit of the propensity score. Covariate balance was assessed using standardized mean differences (SMD), with values < 0.1 indicating acceptable balance.

Stage 2

The second stage evaluated survival in a cohort of patients (*n* = 475) included in 2009. The selection of this subgroup was based on the following criteria to ensure adequate follow-up duration and data completeness for long-term survival assessment:

1. Confirmed AS diagnosis. Patients were required to have a confirmed diagnosis of AS based on TTE findings (Vmax ≥ 2.0 m/s) at the time of inclusion in 2009, ensuring a homogeneous cohort with verified AS for survival analysis.
2. Availability of long-term follow-up data. Patients were included if they had continuous medical records available from 1 January 2009, to 1 January 2025, allowing for a 16-year observation period to assess all-cause mortality using the Kaplan–Meier method.
3. Complete clinical and demographic data. Patients were selected only if they had comprehensive baseline data (age, gender, valve morphology [BAV vs. TAV], comorbidities, and TTE parameters).

Survival was analyzed using the Kaplan–Meier model; missing data were handled by multiple imputation, and matched comparisons used propensity-score matching with post-match balance checks.

Time was measured from the index assessment to death or last contact (all-cause mortality). We assumed independent, non-informative right-censoring. Kaplan–Meier curves with log-rank tests summarized group differences. Continuous covariates were modeled flexibly to satisfy functional-form assumptions. For matched comparisons, propensity-score matching was used and covariate balance verified post-match (standardized mean differences < 0.10). Missing data were handled by multiple imputation under a missing-at-random assumption. Medication exposures were coded as ever/never; because exposures were not time-varying, related findings are interpreted as exploratory.

The survival model was validated based on the patient cohort where the observation started from 2009 (*n* = 475) and ended on 1 January 2025 using the Kaplan–Meier method, which calculates survival as the product of survival probabilities at specific time points when events occur [18].

The study evaluated the incidence of all-cause mortality in patients observed at the Federal State Budgetary Institution “V.A. Almazov National Medical Research Centre” with various diagnoses, subsequently isolating patients with aortic AS and stratifying them by gender and age into the following groups: <60 years, 60–75 years, and 75–90 years.

CAD was diagnosed in the presence of a history of myocardial infarction, as well as in patients with mild and moderate AS and angina symptoms based on stress testing; in patients with severe AS, coronary angiography was performed.

Comorbidities were defined based on ICD codes from the medical records of the patients. The following codes were used:

Hypertension

- I13.X

Diabetes Mellitus

- E10.X
- E11.X

Coronary Artery Disease

- I25.X

Chronic Obstructive Pulmonary Disease

- J44.0—Chronic obstructive pulmonary disease with (acute) lower respiratory infection
- J44.1—Chronic obstructive pulmonary disease with (acute) exacerbation
- J44.9—Chronic obstructive pulmonary disease, unspecified

Asthma

- J45.X

Obesity

- E66.X

Hyperlipidemia

- E78.X

Congestive Heart Failure

- I50.X

*Therapy*

The impact of aortic valve replacement (*n* = 68 operations) on survival was analyzed, without evaluating the temporal factor of mortality onset in relation to the timing of the surgical intervention.

In the analysis of therapy, all medications were categorized into the main known subgroups: angiotensin-converting enzyme inhibitors (ACEi), angiotensin II receptor blockers (ARBs), calcium channel blockers (CCBs), beta-blockers, diuretics, antiarrhythmic medications, antiplatelet medications, anticoagulant medications, lipid-lowering drugs, hypoglycemic drugs, and inotropic agents. Combinations of these medications were also assessed. The dosage and duration of medication use were not analyzed.

*Statistical Methods*

Statistical analysis was performed using STATISTICA v. 10.0 (StatSoft Inc., Tulsa, OK, USA). Given the non-normal distribution of the sample, clinical and demographic characteristics were reported as percentages for qualitative variables and as medians with quartiles [Q1; Q3] for quantitative variables. The P-test was used to assess the distribution of characteristics between groups of patients with BAV and TAV, as well as between male and female patients [STATISTICA, 2010].

*Pre-analysis Data Screening*

Using Tukey’s rule (1.5 × IQR), 11 observations (3.5%) were flagged. Eight were biologically implausible and were winsorized to the 1st/99th percentile; three were retained unchanged after clinical adjudication. Q–Q plots and density overlays confirmed the non-Gaussian distributions suggested by Shapiro–Wilk.

*Missing-data Handling*

- Missingness ranged from 0% to 15.2% per variable (median = 4.3%).
- Little’s test for missing completely at random was significant (χ^2^ = 103.4, df = 78, *p* = 0.018), indicating that the data are not missing completely at random (MCAR).
- Complete-case analysis was used when missingness ≤ 5%
- For variables with 5–20% missingness, we applied multiple imputation by fully conditional specification:
  ○ predictive mean matching (k = 5) for continuous, logistic/multinomial for binary or nominal factors,
  ○ all model covariates and the outcome included,
  ○ convergence inspected via trace plots of means/SDs.
- Any variable with >20% missingness (none in this study) would have been excluded a priori.

## 3. Results

The analysis revealed that men with AS were older and had higher systolic blood pressure (SBP) compared to men without AS (Table 1).

Among comorbidities, men with AS were more frequently diagnosed with HLP, AR, and CHF. Conversely, HP, and obesity were more prevalent in men without verified AS. TTE data showed significantly larger aortic diameters at the sinuses of Valsalva and ascending aorta in men with AS (Table 1).

Female patients with AS were significantly older than women without AS. Similar to men, women with AS had larger ascending aorta diameters by TTE and higher SBP and diastolic blood pressure (DBP) values (Table 2).

CAD, AR, diabetes mellitus (DM), chronic obstructive pulmonary disease (COPD), HLP, and CHF were more frequent in women with AS compared to those without AS. However, like men, women without AS had a higher prevalence of HP (Table 2).

Among male patients, AS was diagnosed in 54.5% of those with BAV (*n* = 536) and 11.1% of those with TAV (*n* = 4423). (Table 3 and Table 4).

Men with AS were older and more frequently diagnosed with CHF, regardless of valve morphology. TTE data showed significantly larger aortic diameters at the sinuses of Valsalva and ascending aorta in men with AS compared to those without AS, regardless of valve morphology (Table 3).

Significant comorbidities in men with BAV and AS included higher rates of CAD, HLP, COPD, CHF, overweight, and obesity. In contrast, men with TAV and AS had significantly higher rates of AR, as well as elevated SBP values (Table 3).

Among female patients, AS was diagnosed in 66.4% of those with BAV (*n* = 365) and 12.7% of those with TAV (*n* = 5928). (Table 4).

Women with AS were older and had a higher prevalence of CHF, regardless of valve morphology. TTE data indicated significantly larger aortic diameters at the ascending aorta in women with AS compared to those without AS, regardless of valve morphology (Table 4).

In contrast to men, women with AS and TAV more frequently exhibited CAD, HLP, overweight, and obesity. Higher SBP and DBP were more prevalent in women with AS and TAV compared to women with TAV without AS (Table 4).

**Figure 2:**
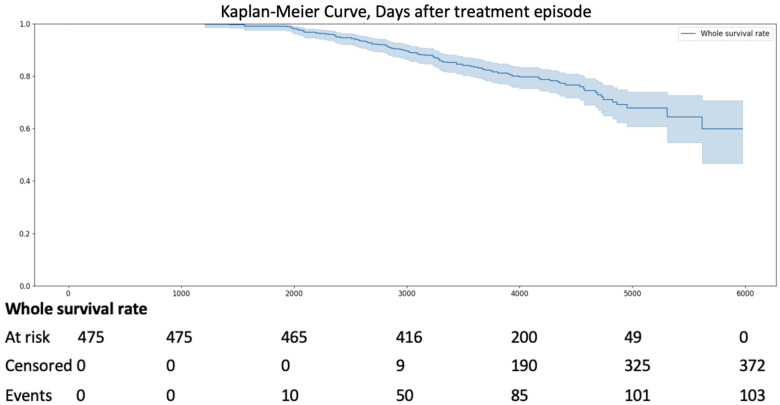
Kaplan–Meier Curve for Survival of Patients with AS (from the year 2009).

Over the 16-year observation period from 2009 to 2025, all-cause mortality occurred in 21.7% of 475 patients with AS of varying severity (*n* = 103) (Figure 2).

**Figure 3:**
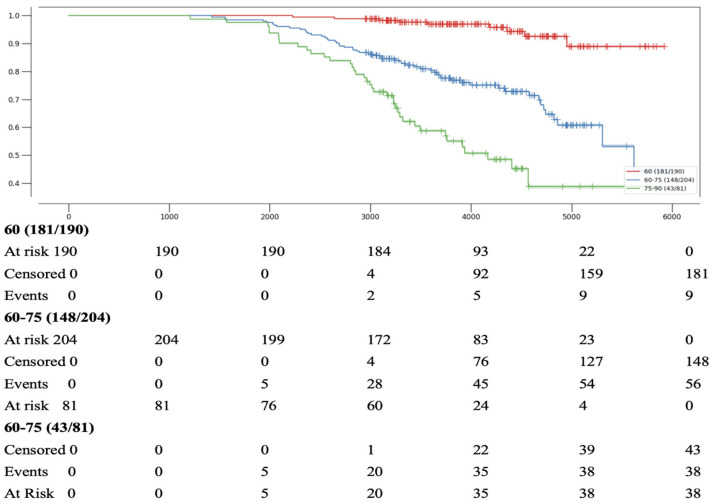
Kaplan–Meier Curves for Survival of Patients Depending on Age.

Age was assessed at the time of inclusion in the study in 2009. Patients were divided into three age groups: under 60 years, 60 to 74 years inclusive, and 75 years and older. The best survival was observed in the group under 60 years: the first fatal events in this group occurred only after 8 years of follow-up and accounted for just 1% of patients in this age category. In comparison, the 8-year mortality rates were 13.7% in the 60–74 age group and 24.7% in the 75+ age group. During longer-term follow-up, most patients were classified as censored, meaning no event was recorded by the end of the observation period (Figure 3).

**Figure 4:**
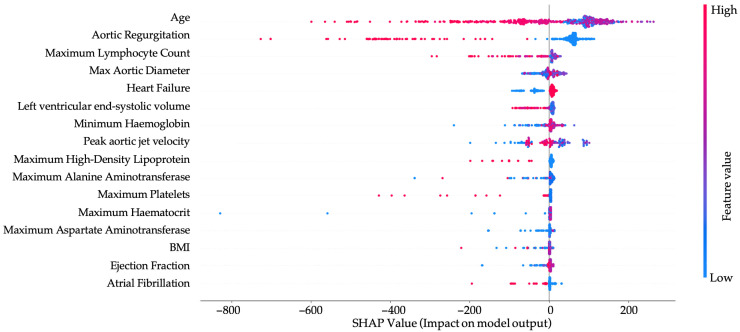
SHAP Summary Plot for Survival Prediction Model. BMI—body mass index.

The SHAP summary plot illustrates the contribution of individual features to the model’s prediction of patient survival (Figure 4).

Age is the strongest predictor, with higher values strongly associated with decreased survival probability. Similarly, the presence of aortic regurgitation, heart failure, peak aortic jet velocity and increased maximum aortic diameter all contribute negatively to predicted outcomes, reflecting their known roles in cardiovascular morbidity and mortality. Lower lymphocyte counts and hemoglobin levels are also associated with worse survival, likely indicating systemic inflammation or anemia-related stress. Conversely, higher values of HDL cholesterol and ejection fraction are generally protective, contributing to improved survival predictions. Structural and functional heart parameters, such as left ventricular end-systolic volume and peak aortic jet velocity, further refine risk stratification by capturing the severity of underlying cardiac disease. Overall, the model captures a coherent clinical picture, with both demographic and pathophysiological markers contributing meaningfully to survival estimation. As already shown in Table 1 and Table 2, patients with AS, both female and male, more frequently presented with the following comorbidities: HLP and CHF. AR was also more commonly diagnosed, along with more pronounced aortic dilatation. According to Figure 4, these comorbid conditions also have an impact on survival in patients with AS.

**Figure 5:**
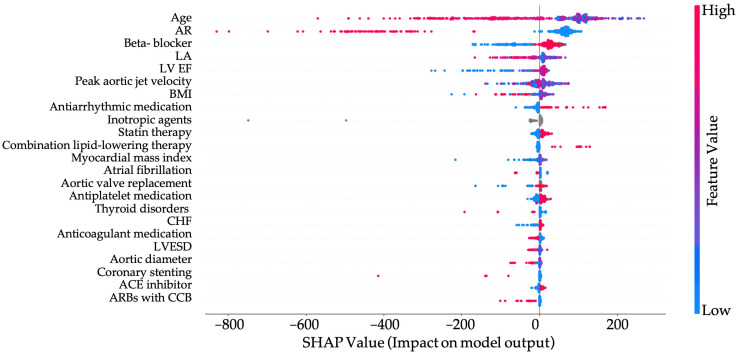
SHAP Summary Plot for Survival Prediction Model including therapy. AR—aortic regurgitation; BMI—body mass index; CHF—chronic heart failure, LA—left atrium, LV—left ventricle; EF—ejection fraction; LVESD—left ventricular end systolic dimension; ARBs with CCB—angiotensin II receptor blockers with Calcium channel blockers.

We analyzed the impact of treatment on patient prognosis, encompassing both conservative and surgical approaches. As previously mentioned, beta-blockers exerted a significant positive effect on the survival of patients with AS, comparable to the absence of aortic regurgitation AR and younger patient age (Figure 5).

According to Figure 5, aortic valve replacement demonstrates an insignificant effect on patient survival rates. Antiarrhythmic medications and lipid-lowering agents, whether used in monotherapy or in combination, were also classified as drugs exerting a positive effect. The absence of AF and anticoagulant medication similarly had a positive impact on outcomes.

**Figure 6:**
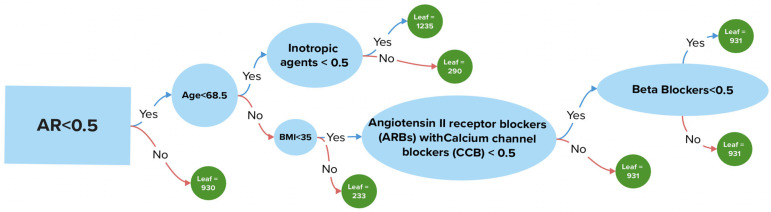
Survival decision tree.

In patients with aortic AR, survival is poorer compared to those without AR. In the absence of AR, survival is influenced by age older than 68.5 years (Figure 6).

In patients older than 68.5 years, a BMI greater than 35 worsened the prognosis.

Conversely, in patients older than 68.5 years but with a BMI less than 35, the prognosis is not similarly affected. If patients do not receive a combination of angiotensin II receptor blockers with CCBs but are treated with beta-blockers, their survival is improved.

To further validate our findings, we conducted a propensity score matching analysis to mitigate potential confounding effects arising from baseline differences between patients with and without AS. After matching, the final analytic cohort included 11,320 patients with AS and an equal number of non-AS controls, achieving well-balanced baseline characteristics across all covariates (all SMD < 0.08). This rigorous matching ensured that comparisons of long-term outcomes were not biased by differences in age, sex, or comorbidities such as hypertension, coronary artery disease, or diabetes mellitus.

Consistent with our main results, all-cause mortality remained significantly higher in the AS group compared to matched controls (Hazard Ratio [HR] 1.35, 95% Confidence Interval [CI] 1.22–1.49, *p* < 0.001). These findings underscore the independent and long-lasting impact of AS on patient survival, even after adjusting for key cardiovascular risk factors.

## 4. Discussion

AS is one of the most common acquired heart valve diseases worldwide [1,2]. Increasingly, publications highlight gender-based differences in AS prevalence and progression [11,12,13]. Our study corroborates these findings and identifies additional gender-specific characteristics.

Historically, AS was considered more prevalent in men [7,9]. However, recent publications suggest a higher detection rate in women, possibly due to improved diagnostic methods and earlier detection [11,19,20]. In our study, female patients with AS (*n* = 6322) outnumbered male patients (*n* = 4998), supporting this trend (Table 1 and Table 2). Our decision to stratify analyses by gender was informed by established sex differences in AS epidemiology and cardiac remodeling [21]. In our previous study on the same population, sex influenced the disease phenotype differently within BAV and TAV AS populations [16]: women with AS were older at presentation and had smaller valve areas, whereas men exhibited greater LV mass and dilatation. Such differences can affect both the interpretation of echocardiographic parameters and clinical decision-making. While improved diagnostics, like echocardiography, may increase AS detection in women, there is a lack of evidence to attribute this solely to detection bias. Anatomical factors, such as smaller aortic roots in women, hormonal influences like estrogen-driven calcification, and higher comorbidity rates may contribute. Future research should explore sex-specific anatomical, hormonal, and genetic differences to clarify AS prevalence in women.

Literature indicates that AS patients frequently have comorbidities such as HP, CAD, CHF, DM, chronic lung diseases, and elevated BMI [22,23,24,25]. Despite timely surgical intervention, CHF prevalence remains high in AS patients and is strongly associated with poorer prognosis [26]. Our analysis confirmed a higher CHF prevalence in AS patients, with 53.4% of men and 54.5% of women affected (Table 1, Table 2, Table 3 and Table 4).

In 2018, the ARIC (Atherosclerosis Risk in Communities) study reported a higher CAD prevalence in AS patients [27]. Our findings align with this, with CAD being significantly more common in men with AS and BAV compared to those without AS, and also in men with AS and TAV, in contrast to patients with TAV without AS (Table 3).

Over 60% of AS patients have concomitant HP, which accelerates valve pathology progression and worsens prognosis [27,28]. In our cohort, HP and elevated SBP and DBP were significantly more prevalent in women with AS and TAV compared to those without AS (Table 4).

Currently, no pharmacological agents have demonstrated proven efficacy in preventing the progression of AS. AS itself does not have an indication for specific therapeutic interventions according to clinical guidelines. However, patients with AS frequently present with comorbidities or complications, such as arterial hypertension or arrhythmias, which necessitate targeted therapies.

The potential use of ACEi or ARBs in AS management has been extensively debated. According to current clinical guidelines, these drug classes are permitted in patients with AS and concomitant HP. Regarding CCBs, their use in severe AS is contraindicated, per the medication package insert. However, in the present study, some patients with mild to moderate AS received CCBs, and these were not consistently discontinued upon AS progression. Notably, this did not significantly impact survival, unlike specific combinations of CCBs with ARBs. The absence of a negative impact on survival and complications in patients with severe AS receiving CCB monotherapy was previously demonstrated by Yamamoto et al., who reported that syncope was rarely observed in patients with severe AS and HP receiving antihypertensive therapy, irrespective of CCB use [29]. Conversely, earlier studies, such as Saeed et al., reported adverse effects, including reduced treadmill exercise capacity and decreased survival in asymptomatic patients with moderate to severe AS receiving CCBs [30].

Thus, the impact of combined CCB and ARB therapy, as well as CCB monotherapy, on survival and complications in AS patients warrants further in-depth investigation.

The prescription of beta-blockers in patients with AS and concomitant HP is generally avoided due to concerns about reducing left ventricular (LV) function. However, in the present study, beta-blockers not only showed no adverse effects but also demonstrated a positive impact on survival in AS patients older than 68 years. This effect may be attributed to their beneficial influence on concomitant CAD and arrhythmias, the prevalence of which increases with age. The absence of a negative effect of beta-blockers in AS patients was previously supported by Bang et al., who showed that beta-blocker therapy did not increase the risk of all-cause mortality, sudden cardiac death, or cardiovascular death in patients with asymptomatic mild to moderate AS [31].

Although two large, randomized trials previously demonstrated the ineffectiveness of statin therapy in slowing AS progression in patients with severe stage AS, research on lipid-lowering therapy in AS patients with mild and moderate stage AS continues. The present study observed a positive effect of statins on survival, which may be related to beneficial effects on both AS and concomitant CAD [32,33].

Comorbidities such as diabetes significantly affect outcomes in severe AS. In our cohort, diabetes was more common in AS patients than controls (13.4% vs. 10.2%). Recent studies [33,34] show that SGLT2 inhibitors in diabetic patients reduce mortality, heart failure hospitalizations, and post-TAVI AKI. Moreover, emerging evidence suggests that SGLT2 inhibitors (gliflozins) may have prognostic benefits in patients with structural heart conditions, including aortic stenosis [35]. Although our patients did not receive gliflozins in this study, this is a very interesting suggestion, and we plan to consider their use in prospective studies in AS patients.

Although our study focused on valve pathology and survival, these findings suggest that managing diabetes and preserving renal function could further improve long-term prognosis alongside valve intervention. The observed survival benefit with beta-blockers in our results also highlights the role of optimal medical therapy in this population.

The association between elevated BMI and AS has yielded conflicting results in prior studies [36,37,38,39]. A large genomic meta-analysis of 13,765 AS patients confirmed a genetic link between HLP, overweight, and obesity with AS [40]. Some studies suggest that women with overweight or obesity are diagnosed with AS three times more frequently than those with normal BMI [39]. Our data showed that HLP, overweight, and obesity were significantly more common in women with AS compared to women without AS, highlighting the need for further gender-specific research (Table 2 and Table 4).

TTE revealed larger aortic diameters at the sinuses of Valsalva and ascending aorta in AS patients, regardless of gender or valve morphology, consistent with existing literature [41].

Several studies report worse survival outcomes in women with AS compared to men, potentially due to later diagnosis in women [11,42,43]. Our study confirmed this trend, with higher mortality rates in women, underscoring the importance of gender-specific approaches to improve clinical outcomes.

## 5. Clinical Implications

Current ESC/EACTS and ACC/AHA guidelines do not provide a specific recommendation for routine beta-blocker therapy directed at aortic stenosis; beta-blockers may be used for standard indications (e.g., rate control, ischemia, hypertension) with careful titration. By contrast, ACE inhibitors or ARBs are considered acceptable for treating concomitant hypertension or heart failure in patients with AS. In line with this framework, our cohort showed an association between beta-blocker therapy and improved survival, which we interpret as supportive of GDMT (e.g., control of ischemia/arrhythmias) rather than a change in indications. ACC/AHA guidance notes that medical treatment of hypertension/hyperlipidemia is appropriate in AS and that RAS inhibition after TAVI may be considered to reduce long-term all-cause mortality (Class IIb, Level B). Our exploratory signal of harm with combined ARB + CCB therapy warrants cautious interpretation given possible confounding and immortal-time bias and should prompt medication review rather than a change in practice. The age cut-point (68.5 years) identifies higher-risk patients for closer follow-up but does not constitute a management threshold, consistent with guideline criteria that rely on symptoms, LV function, and hemodynamics.

## 6. Limitations

This study provides valuable insights into the gender-specific characteristics of patients with AS; however, several limitations should be acknowledged. First, the retrospective nature of the Stage 1 analysis, which relied on echocardiographic data from 2009 to 2018, may introduce selection bias and limit the generalizability of findings due to potential inconsistencies in data collection or documentation over time. Second, the sample size for the survival analysis in Stage 2 was not fully specified, which may affect the statistical power and precision of mortality estimates. Third, the study period extending to 2025 includes future projections, which introduces uncertainty as data beyond January 2025, were not available at the time of analysis. Fourth, while the study adjusted for numerous clinical and demographic variables, unmeasured confounders, such as socioeconomic factors, lifestyle variables, or genetic predispositions beyond bicuspid aortic valve status, could influence the observed outcomes. Fifth, the reliance on TTE for AS diagnosis may be subject to inter-observer variability, and advanced imaging modalities, such as cardiac MRI or CT, were not utilized to corroborate findings. The reasons for lethal outcomes were not analyzed, nor was therapy evaluated based on drug dosages. No data are available regarding the time interval between the operation and the occurrence of lethal outcomes.

Our survival analyses assume independent, non-informative censoring. Medication exposures were coded as non-time-varying, and treatment allocation was non-random; therefore, associations between therapies and mortality are vulnerable to immortal-time and confounding-by-indication biases and should be interpreted as exploratory. Cause-specific mortality was unavailable; we therefore analyzed all-cause mortality and did not perform competing-risks models.

Finally, the study was conducted at a single center, which may limit its applicability to diverse populations with varying healthcare access and AS prevalence. These limitations highlight the need for prospective, multicenter studies to validate and expand upon our findings.

## 7. Conclusions

This study provides a comprehensive analysis of clinical and demographic characteristics, comorbidities, and long-term outcomes in patients with AS. The findings highlight a high burden of comorbid conditions such as HLP and AR in patients with AS, irrespective of valve morphology. Echocardiographic data further revealed significant differences in aortic dimensions and hemodynamic parameters between AS and non-AS patients. Over a 16-year follow-up period, all-cause mortality in a representative AS cohort reached 21.7%, underscoring the progressive nature of the disease and its long-term impact. These results emphasize the importance of early identification, comprehensive risk assessment, and individualized management strategies in improving outcomes for patients with AS. Survival was negatively affected by age over 68.5 years, as well as the presence of AR increased peak aortic jet velocity and enlarged maximum aortic diameter. Beta-blocker therapy in patients with varying degrees of aortic AS severity has not only demonstrated its safety but also shown a positive effect on reducing mortality (improving survival). In contrast, the combination of angiotensin II receptor blockers (ARBs) with calcium channel blockers (CCBs) is quite dangerous for patients with AS and reduces their survival. Aortic valve replacement demonstrates an insignificant effect on patient survival rates. In contrast, the absence of fibrinolytic therapy and anticoagulant treatment is associated with an improved prognosis. Conversely, the administration of antiarrhythmic agents and statins is correlated with enhanced survival outcomes, potentially attributable to their influence on coexisting comorbidities. Further research is required to delineate their precise mechanisms and contributions.

## 8. Highlights

This study provides new insights into the factors influencing long-term outcomes in patients with aortic stenosis AS. The analysis identifies key clinical predictors of survival, as well as treatment regimens that may either improve or worsen prognosis, underscoring the importance of tailored therapeutic approaches in AS management.

- Survival in patients with aortic stenosis was significantly reduced in those aged >68.5 years and in the presence of concomitant aortic regurgitation (AR).
- Beta-blocker therapy was associated with improved survival, demonstrating a protective effect in patients with varying AS severity.
- The combination of angiotensin II receptor blockers and calcium channel blockers was linked to poorer survival outcomes and should be used with caution in patients with AS.

## Figures and Tables

**Table 1:** Clinical and Demographic Characteristics of Male Patients Undergoing Echocardiography at the Federal State Budgetary Institution “V.A. Almazov National Medical Research Centre”, Depending on the Presence or Absence of Aortic Stenosis.

Parameter	Men with AS (*n* = 4998)	Men Without AS (*n* = 35,690)	*p*-Value
Age, years, Me [Q1; Q3]	64 [55; 74]	57 [45; 65]	<0.0001
Aortic diameter at sinuses of Valsalva, mm, Me [Q1; Q3]	37 [34; 39]	36 [33; 39]	<0.0001
Ascending aorta diameter, mm, Me [Q1; Q3]	37 [34; 41]	34 [31; 37]	<0.0001
BMI, kg/m^2^, Me [Q1; Q3]	27 [24; 30]	27 [25; 31]	0.23
Peak aortic jet velocity, m/s, Me [Q1; Q3]	2.74 [2.3; 3.7]	1.6 [1.3; 1.13]	<0.0001
Peak aortic valve gradient, mmHg, Me [Q1; Q3]	30 [20; 53]	6 [5; 8]	<0.0001
LV EF, %, Me [Q1; Q3]	60 [51; 65]	58 [48; 64]	<0.0001
Office SBP, mmHg, Me [Q1; Q3]	140 [127; 150]	130 [120; 140]	<0.0001
Office DBP, mmHg, Me [Q1; Q3]	80 [80; 88]	80 [80; 88]	0.31
AR, *n* (%)	901 (18.03)	1405 (3.94)	<0.0001
HP, *n* (%)	3150 (63.03)	28,920 (81.03)	<0.0001
DM, *n* (%)	485 (9.7)	3218 (9.02)	0.11
CAD, *n* (%)	1963 (39.3)	14,250 (39.9)	0.38
COPD, *n* (%)	521 (10.2)	3707 (10.4)	0.93
Asthma, *n* (%)	110 (2.2)	741 (2.08)	0.56
Obesity, *n* (%)	417 (8.3)	4581 (12.8)	<0.0001
HLP, *n* (%)	1313 (26.3)	3685 (10.3)	<0.0001
CHF, *n* (%)	2671 (53.4)	14,908 (41.8)	<0.0001

BMI—body mass index; LV—left ventricle; EF—ejection fraction; SBP—systolic blood pressure; DBP—diastolic blood pressure; AR—aortic regurgitation; AS—aortic stenosis. COPD—chronic obstructive pulmonary disease; CAD—coronary artery disease, HLP—hyperlipidemia; CHF—chronic heart failure; DM—Diabetes mellitus; HP—Hypertension.

**Table 2:** Clinical and Demographic Characteristics of Female Patients Undergoing Echocardiography at the Federal State Budgetary Institution “V.A. Almazov National Medical Research Centre”, Depending on the Presence or Absence of Aortic Stenosis.

Parameter	Women with AS (*n* = 6322)	Women Without AS (*n* = 37,841)	*p*-Value
Age, years, Me [Q1; Q3]	70 [61; 76]	58 [41; 68]	<0.0001
Aortic diameter at sinuses of Valsalva, mm, Me [Q1; Q3]	32 [30; 35]	32 [30; 34]	<0.0001
Ascending aorta diameter, mm, Me [Q1; Q3]	34 [32; 37]	31 [28; 34]	<0.0001
BMI, kg/m^2^, Me [Q1; Q3]	29 [25; 32]	27 [23; 31]	<0.0001
Peak aortic jet velocity, m/s, Me [Q1; Q3]	2.8 [2.3; 3.9]	1.35 [1.2; 1.5]	<0.0001
Peak aortic valve gradient, mmHg, Me [Q1; Q3]	32 [20; 60]	7 [5; 9]	<0.0001
LV EF, %, Me [Q1; Q3]	62 [58; 65]	63 [58; 65]	0.37
Office SBP, mmHg, Me [Q1; Q3]	140 [125; 150]	130 [120; 140]	<0.0001
Office DBP, mmHg, Me [Q1; Q3]	80 [80; 90]	80 [75; 86]	<0.0001
AR, *n* (%)	880 (13.9)	1274 (3.4)	<0.0001
HP, *n* (%)	3794 (60.01)	26,996 (71.3)	<0.0001
DM, *n* (%)	848 (13.4)	3875 (10.2)	<0.0001
CAD, *n* (%)	2254 (35.7)	9973 (26.4)	<0.0001
COPD, *n* (%)	445 (7.04)	2145 (5.7)	<0.0001
Asthma, *n* (%)	218 (3.5)	1138 (3.01)	0.06
Obesity, *n* (%)	694 (10.9)	3863 (10.2)	0.06
HLP, *n* (%)	1763 (27.9)	8901 (23.5)	<0.0001
CHF, *n* (%)	3445 (54.5)	14,170 (37.5)	<0.0001

BMI—body mass index; LV—left ventricle; EF—ejection fraction; SBP—systolic blood pressure; DBP—diastolic blood pressure; AR—aortic regurgitation; AS—aortic stenosis. COPD—chronic obstructive pulmonary disease; CAD—coronary artery disease, HLP—hyperlipidemia; CHF—chronic heart failure; DM—Diabetes mellitus; HP—Hypertension.

**Table 3:** Clinical and Demographic Characteristics of Male Patients Undergoing Echocardiography at the Federal State Budgetary Institution “V.A. Almazov National Medical Research Centre”, Depending on Aortic Valve Morphology.

Parameter	Men with BAV, AS (*n* = 536)	Men with BAV, no AS (*n* = 447)	*p*-Value	Men with TAV, AS (*n* = 4423)	Men with TAV, no AS (*n* = 35,280)	*p*-Value
Age, years, Me [Q1; Q3]	50 [34; 60]	29 [21; 46]	<0.0001	66 [57; 74]	57 [46; 65]	<0.0001
Aortic diameter at sinuses of Valsalva, mm, Me [Q1; Q3]	37 [34; 41]	37 [33; 41]	<0.05	36 [34; 39]	36 [33; 39]	<0.0001
Ascending aorta diameter, mm, Me [Q1; Q3]	39 [35; 44]	24.8 [21.8; 27.8]	<0.0001	37 [34; 40]	34 [31; 37]	<0.0001
BMI, kg/m^2^, Me [Q1; Q3]	26.3 [23.9; 30]	24.8 [21.8; 27.8]	<0.0001	27.3 [24.5; 30.3]	27.4 [24.5; 30.6]	0.48
Peak aortic jet velocity, m/s, Me [Q1; Q3]	2.7 [2.3; 3.5]	1.6 [1.4; 1.8]	<0.0001	2.75 [2.3; 3.7]	1.25 [1.1; 1.4]	<0.0001
Peak aortic valve gradient, mmHg, Me [Q1; Q3]	29 [20; 50]	10 [7; 12]	<0.0001	30 [20; 53]	6 [5; 8]	<0.0001
LV EF, %, Me [Q1; Q3]	63.8 [57.4; 69]	64.2 [59.5; 69.7]	0.04	62.4 [53.4; 68]	60.9 [51.5; 67]	<0.0001
Office SBP, mmHg, Me [Q1; Q3]	135 [124; 142]	130 [120; 140]	0.12	140 [129; 150]	130 [120; 140]	<0.0001
Office DBP, mmHg, Me [Q1; Q3]	80 [75; 85]	80 [80; 85]	0.86	80 [80; 88]	80 [80; 87]	0.46
AR, *n* (%)	123 (22.95)	118 (26.4)	0.20	778 (17.59)	1286 (3.65)	<0.0001
HP, *n* (%)	316 (58.95)	268 (59.96)	0.5	2803 (63.37)	25,532 (72.37)	<0.001
DM, *n* (%)	31 (5.78)	17 (3.80)	0.15	451 (10.20)	3204 (9.08)	0.2
CAD, *n* (%)	127 (23.69)	46 (10.29)	<0.001	1818 (41.10)	14,222 (40.31)	0.31
COPD, *n* (%)	58 (10.82)	23 (5.15)	<0.001	460 (10.40)	3687 (10.45)	0.92
Asthma, *n* (%)	22 (4.10)	9 (2.01)	0.06	88 (1.99)	732 (2.07)	0.71
Obesity, *n* (%)	61 (11.3)	19 (4.25)	<0.0001	394 (8.9)	3005 (8.52)	0.15
HLP, *n* (%)	135 (25.19)	56 (12.53)	<0.001	1170 (26.45)	9061 (25.68)	0.27
CHF, *n* (%)	320 (59.7)	156 (34.90)	<0.001	2332 (52.72)	14,770 (41.87)	<0.001

BAV—Bicuspid Aortic Valve; TAV—tricuspid aortic valve; BMI—body mass index; LV—left ventricle; SBP—systolic blood pressure; DBP—diastolic blood pressure; AR—aortic regurgitation; AS—aortic stenosis. COPD—chronic obstructive pulmonary disease; CAD—coronary artery disease, HLP—hyperlipidemia; CHF—chronic heart failure; DM—Diabetes mellitus; HP—Hypertension.

**Table 4:** Clinical and Demographic Characteristics of Female Patients Undergoing Echocardiography at the Federal State Budgetary Institution “V.A. Almazov National Medical Research Centre”.

Parameter	Women with BAV, AS (*n* = 365)	Women with BAV, no AS (*n* = 185)	*p*-Value	Women with TAV, AS (*n* = 5928)	Women with TAV, no AS (*n* = 40,925)	*p*-Value
Age, years, Me [Q1; Q3]	49 [31; 61]	31 [26; 49]	<0.0001	71 [62; 77]	58 [41; 68]	<0.0001
Aortic diameter at sinuses of Valsalva, mm, Me [Q1; Q3]	32 [30; 35]	32 [29; 36]	0.89	32 [30; 35]	32 [30; 34]	<0.0001
Ascending aorta diameter, mm, Me [Q1; Q3]	36 [32; 40]	33 [29; 39]	<0.0001	34 [31; 37]	31 [28; 34]	<0.0001
BMI, kg/m^2^, Me [Q1; Q3]	25.6 [22.6; 29.9]	24.6 [21.7; 26.9]	0.01	28.8 [25.2; 32.6]	27.1 [23.5; 31.2]	<0.0001
Peak aortic jet velocity, m/s, Me [Q1; Q3]	2.9 [2.4; 3.8]	1.62 [1.4; 1.8]	<0.001	2.8 [2.3; 3.9]	1.62 [1.4; 1.8]	<0.001
Peak aortic valve gradient, mmHg, Me [Q1; Q3]	32 [22; 56]	10 [8; 13]	<0.0001	31 [20; 60]	7 [5; 9]	<0.0001
LV EF, %, Me [Q1; Q3]	66.9 [61.8; 71.4]	66 [61; 70]	0.15	65.9 [60.7; 70]	65.7 [60.6; 70]	0.34
Office SBP, mmHg, Me [Q1; Q3]	120 [120; 140]	120 [110; 127.5]	0.13	140 [130; 150]	130 [120; 140]	<0.0001
Office DBP, mmHg, Me [Q1; Q3]	80 [70; 80]	80 [70; 80]	0.61	80 [80; 90]	80 [75; 85]	<0.0001
AR, *n* (%)	64 (17.53)	27 (14.59)	0.38	814 (13.74)	1284 (3.14)	<0.0001
HP, *n* (%)	177 (48.5)	99 (53.5)	0.1	3589 (60.5)	26,923 (65.78)	0.3
DM, *n* (%)	20 (5.48)	9 (4.86)	0.76	824 (13.90)	3870 (9.45)	0.1
CAD, *n* (%)	58 (15.89)	18 (9.73)	0.05	2183 (36.83)	9968 (24.35)	<0.001
COPD, *n* (%)	15 (4.11)	5 (2.70)	0.4	426 (7.19)	2144 (5.23)	0.5
Asthma, *n* (%)	10 (2.74)	5 (2.70)	0.98	208 (3.51)	1133 (3.01)	0.8
Obesity, *n* (%)	47 (12.8)	5 (2.70)	0.002	862 (14.54)	4026 (9.83)	<0.01
HLP, *n* (%)	94 (25.75)	19 (10.27)	<0.001	1661 (28.02)	8888 (21.71)	<0.01
CHF, *n* (%)	94 (62.7)	179 (44.8)	0.002	1451 (53.9)	15,891 (38.8)	<0.001

BAV—Bicuspid Aortic Valve; TAV—tricuspid aortic valve; BMI—body mass index; LV—left ventricle; SBP—systolic blood pressure; DBP—diastolic blood pressure; AR—aortic regurgitation; AS—aortic stenosis. COPD—chronic obstructive pulmonary disease; CAD—coronary artery disease, HLP—hyperlipidemia; CHF—chronic heart failure; DM—Diabetes mellitus; HP—Hypertension.

## Data Availability

The data presented in this study are available on request from the corresponding author.

## Abbreviations

HP	hypertension
AV	aortic valve
AR	aortic regurgitation
AS	aortic stenosis
BAV	bicuspid aortic valve
HLP	hyperlipidemia
DBP	diastolic blood pressure
CAD	coronary artery disease
BMI	body mass index
LV	left ventricle
LA	left atrium
LVESD	left ventricular end systolic dimension
SBP	systolic blood pressure
DM	diabetes mellitus
TAV	tricuspid aortic valve
TTE	transthoracic echocardiography
EF	ejection fraction
COPD	chronic obstructive pulmonary disease
CHF	chronic heart failure
